# Variable stretch reduces the pro-inflammatory response of alveolar epithelial cells

**DOI:** 10.1371/journal.pone.0182369

**Published:** 2017-08-15

**Authors:** Ines Rentzsch, Cíntia L. Santos, Robert Huhle, Jorge M. C. Ferreira, Thea Koch, Christian Schnabel, Edmund Koch, Paolo Pelosi, Patricia R. M. Rocco, Marcelo Gama de Abreu

**Affiliations:** 1 Department of Anesthesiology and Intensive Care Therapy, Pulmonary Engineering Group, University Hospital Carl Gustav Carus, Technische Universität Dresden, Dresden, Germany; 2 Laboratory of Pulmonary Investigation, Carlos Chagas Filho Institute of Biophysics, Federal University of Rio de Janeiro, Rio de Janeiro, Brazil; 3 Department of Surgical Sciences and Integrated Diagnostics, AOU IRCCS San Martino –IST, University of Genoa, Genoa, Italy; Universitatsklinikum Freiburg, GERMANY

## Abstract

Mechanical ventilation has the potential to increase inflammation in both healthy and injured lungs. Several animal studies have shown that variable ventilation recruits the lungs and reduces inflammation. However, it is unclear which cellular mechanisms are involved in those findings. We hypothesized that variable stretch of LPS-stimulated alveolar epithelial cells (AECs) reduces the production of pro-inflammatory cytokines compared to non-variable stretch. AECs were subjected to non-variable or variable cyclic stretch (sinusoidal pattern), with and without LPS stimulation. The expression and release of interleukin-6, CXCL-2 and CCL-2 mRNA were analyzed after 4 hours. The phosphorylation of the MAPKs ERK1/2 and SAPK/JNK was determined by Western Blot analysis at 0, 15, 30, 45 and 60 min of cyclic stretch. In LPS-stimulated AECs, variable cyclic cell stretching led to reduced cytokine expression and release compared to non-variable cell stretching. Furthermore, the phosphorylation of the MAPK ERK1/2 was increased after 30 minutes in non-variable stretched AECs, whereas variable stretched cells demonstrated only the non-stretched level of phosphorylation. After the 4h period of cyclic cell stretch and inhibition of the ERK1/2, but not the SAPK/JNK, signaling pathway, the gene expression of investigated cytokines increased in variable stretched, and decreased in non-variable stretched AECs. We conclude that in LPS-stimulated AECs, variable stretch reduced the pro-inflammatory response compared to non-variable stretch. This effect was mediated by the ERK1/2 signaling pathway, and might partly explain the findings of reduced lung inflammation during mechanical ventilation modes that enhance breath-by-breath variability of the respiratory pattern.

## Introduction

The use of breath-by breath variable tidal volumes during controlled mechanical ventilation (MV), mimicking some of the properties of spontaneous breathing, has emerged as a promising approach in protective ventilation. In animal models of the acute respiratory distress syndrome (ARDS), variable as compared to non-variable tidal volumes improved lung function [1, 2], reduced histological damage [2] and decreased interleukin (IL)-8 concentrations in bronchoalveolar lavage fluid [1]. Such effects are mediated by different mechanisms, including recruitment of lung units with more favorable distribution of stress-and-strain [3], redistribution of perfusion [2, 4], and increased release of lung surfactant [5]. Theoretically, a differentiated molecular biological response to variable cell stretch might also partly explain those effects.

In alveolar epithelial cells (AECs), non-variable cyclic stretch, as compared to non-stretched resting conditions, induces the production of cytokines [6, 7] and reactive oxygen species [8], increases cell permeability [9], and promotes remodeling of the cytoskeleton [10]. Those effects are mediated by different specific signaling pathways, including those dependent on mitogen-activated protein kinase (MAPK) [11]. The MAPK family is a highly conserved family of serine-threonine-kinases consisting of three main members, namely extracellular-signal related kinase 1/2 (ERK1/2), stress-activated protein kinase (SAPK/JNK) and p38. They can be activated by growth factors [12], oxidative stress [13] but also strain [14], transferring environmental mechanical stimuli to the nucleus. In rats, high pressure ventilation activates ERK1/2 and SAPK pathways [15]. *In vitro* studies on AECs revealed that non-variable cyclic stretch results in the activation of the several MAPK pathways [16–18]. To our knowledge, however, the mechanisms involved in the response of AECs to variable cyclic stretch have not been determined. In the present study, we tested the hypothesis that variable cyclic stretch of AECs reduces the release of pro-inflammatory cytokines compared to non-variable cyclic stretch via different MAPK pathways.

## Material and methods

### Cell culture and stretch

Experiments were conducted in two types of alveolar epithelial cells (AECs) from rats, namely the L2 cell line (ATCC, Wesel, Germany), and primary type I-like AECs. L2 AECs were cultured in DMEM (Biochrom, Berlin, Germany) containing 10% FBS and 50 μg gentamycin sulfate/ml (Biochrom), seeded on silicon collagen I-coated BioFlex six-well plates (Flexcell International Corporation, Hillsborough, USA) at a density of 1.2 x 10^5^ cells/well, and cultured at 37°C in a humidified atmosphere with 6.5% CO_2_ for 36h. Following approval by the Landesdirektion Sachsen, Referat 24.2 (permit no. DD24-5131/365/2), primary type II AECs were isolated from male Wistar-Han rats (180–210 g) using a technique adapted from Gonzalez et al. [19] Briefly, after anesthesia with an i.p. injection of Midazolam (2 mg/kg/body weight, Hameln pharma plus GmbH, Hameln, Germany), Ketamin (200 mg/kg/body weight, Inresa Arzneimittel GmbH, Freiburg, Germany) and Heparin (2500 I.E./kg/body weight, Rotexmedica, Trittau, Germany), the abdominal aorta was descended, the trachea cannulated and through the pulmonary artery the lungs were rinsed with 10 ml F-12K (ATCC, Wesel, Germany) supplemented with 25 mM HEPES (SIGMA-Aldrich, St. Louis, USA) to remove the blood. The lungs were surgically removed and lavaged 5 times with phosphate buffered saline supplemented with 5mM ethylenediaminetetraacetic acid (EDTA, SIGMA-Aldrich) and 5 mM ethylene glycol-bis (β-aminoethylether)-N,N’,N’-tetraacetic acid (EGTA, SIGMA-Aldrich). Upon instillation of 40 ml warm F-12K supplemented with 50 mM HEPES and 14.3 units/ml Elastase (Worthington, Lakewood, New Jersey, USA), 2.5 mg Dispase (SIGMA-Aldrich) and 20 mg Trypsin (SIGMA-Aldrich) within 15 min, the lungs were incubated for 45 min at 37°C. Following digestion, the lungs were minced and incubated with 20 ml of F-12K supplemented with 25 mM HEPES, 20% FBS and 0.2 ml DNase (5 mg/ml, SIGMA-Aldrich) for 5 min at 37°C by shaking. The sample was then filtered through progressively finer meshes of cotton gauze (Fink & Walter, Merchweiler, Germany) following filtration through nylon mesh (20 μM, Heidland gmbH & Co KG, Harsewinkel, Germany). Upon centrifugation of the cell suspension at 150 x g for 10 min and resuspension in DMEM (Biochrom, Berlin, Germany) the cells were incubated for 1h on plastic plates covered with rat IgG (SIGMA-Aldrich) for 1h at 37°C. The nonadherent cells were removed and centrifuged at 150 x g for 10 min and resuspended in DMEM supplemented with 10% FBS and 50 μg gentamycin sulfate/ml (Biochrom). Cells were seeded at a density of 2x10^5^ cells/well onto silicon collagen I-coated BioFlex Six-Well plates (Flexcell International Corporation, Hillsborough, USA) and cultured for 5 days at 37°C in a humidified atmosphere with 6.5% CO_2_ in DMEM with 10% FBS and 50 μg gentamycin sulfate/ml, yielding type I-like AECs (S1 Fig) [9, 19]. 16–20 h before stretch experiments, L2 and type I-like AECs were incubated with DMEM containing 1% FBS and 50 μg gentamycin sulfate/ml.

Plates were randomly assigned to non-variable (peak 7.5%), or variable (random variable peaks between 1 and 15%, mean 7.5%, normal distribution, coefficient of variation 30%) stretching for different periods depending of method. The stretching frequency was 0.5 Hz, and the stretching/relaxation ratio was 1:1 (sinusoidal pattern). Plates were further randomized for preincubation with either pro-inflammatory priming with LPS (*Escherichia coli* O111:B4, 1h, 2 μg/ml, SIGMA-Aldrich, St.Louis, USA), the MEK1/2 inhibitor IV (PD184161, 2μM, 30 min, Calbiochem, Darmstadt, Germany; upstream kinase of ERK1/2), the JNK inhibitor II (SP 600125, 20 μM, 1h, Calbiochem, Darmstadt, Germany).

#### Stretching device

The custom designed device was constructed to stretch three wells of a BioFlex Culture six-well plate (Flexcell International Corporation, Hillsborough, USA) simultaneously from the back side of the silicone membranes (S2 Fig). The stretch of the membranes is performed by three indenters that are in contact with the membranes. The vertical displacement of the cylindrical indenters results in a homogeneous stretch of each membrane. The three intenders are attached on a plate under the BioFlex Culture wells. Vertical motion of the intender plate is performed by a stepper motor (Maxon Motor AG, Sachseln, Switzerland). A custom made program controls the driving stepper motor and allows the adjustment of the stretching parameters like frequency and stretching amplitude (S3 Fig).

### Cell viability assay

The Cytotoxicity Detection Kit Plus (Roche, Mannheim, Germany) was used to determine lactate dehydrogenase (LDH) levels and assess cell viability.

### Quantification of RNA

Total RNA was extracted using the NucleoSpin RNA II Kit (Macherey & Nagel, Düren, Germany) and reverse transcribed with the RevertAid H Minus First Strand cDNA Synthesis Kit (Thermo Scientific, Waltham, USA). For Real Time PCR, the Maxima SYBR Green qPCR Mastermix (Thermo Scientific) was used. cDNA products were analyzed by semiquantitative RT-PCR using the ΔΔCT method. GAPDH and HPRT served as housekeeping genes. Primers used are listed in Table 1.

**Table 1:** Primers for Real Time PCR purchased from MWG (Ebersberg, Germany).

Primer	Sequence (5’-3’)	Length of cDNA product	Transcriptnumber in Ensembl
GAPDH s	AAC TTT GGC ATC GTG GAA GGG CT	138 bp	ENSRNOT00000050443
GAPDH as	ACC AGT GGA TGC AGG GAT GAT GTT		
HPRT s	TTT CCT TGG TCA AGC AGT ACA GCC C	89 bp	ENSRNOT00000045153
HPRT as	TGG CCT GTA TCC AAC ACT TCG AGA		
IL-6 s	GAC AAA GCC AGA GTC ATT CAG AG	165 pb	ENSRNOT00000013732
IL-6 as	TTG GAT GGT CTT GGT CCT TAG CC		
IL-4 s	CGG TCT GAA CTC ACT GAG AAG	104 bp	ENSRNOT00000010029
IL-4 as	GCA AGT ATT TCC CTC GTA GGA T		
IL-10 s	TGA ATT CCC TGG GAG AGA AGC TGA	107 bp	ENSRNOT00000006246
IL-10 as	ATT CTT CAC CTG CTC CAC TGC CTT		
CXCL-2 s	AGA ACA TCC AGA GCT TGA CGG TG	108 bp	ENSRNOT00000003745
CXCL-2 as	GGG CTT CAG GGT TGA GAC AAA CT		
CCL-2 s	ATG ATC CCA ATG AGT CGG CTG GAG	104 bp	ENSRNOT00000009448
CCL-2 as	GCA CAG ATC TCT CTC TTG AGC TTG G		

### Western Blot analysis

Equal amounts of protein extracts were separated on SDS gels, transferred to PVDF membranes (Millipore, Darmstadt, Germany) using a tank blotting system (BioRad, Munich, Germany) and incubated with primary antibody (Table 2) over night at 4°C. Secondary HRP-conjugated goat anti-mouse (Cell Signaling) or goat anti-rabbit (BioRad) immunoglobulins were used at a dilution of 1:4000. The Blots were developed with Western Bright Chemo-luminescence Substrate Quantum (Advansta, Menlo Park, USA) and visualized employing a LAS 3000 (FujiFilm, Tokyo, Japan). Densitometric evaluation of the protein bands was performed using ImageJ software and normalized with GAPDH. MAPK fold-change was calculated relative to unstimulated non-stretched samples at zero min.

**Table 2:** Details and incubation protocols of the used antibodies.

Antibody	Isotype	Producer	Incubation protocol
pSAPK	rabbit monoclonal	Cell Signaling (Leiden, Netherlands)	1:1000 in PBS containing 5% BSA
pERK1/2	mouse monoclonal	Cell Signaling (Leiden, Netherlands)	1:4000 in PBS containing 5% BSA
pFAK576	rabbit polyclonal	Cell Signaling (Leiden, Netherlands)	1:1000 in PBS containing 5% BSA
GAPDH	mouse monoclonal	SIGMA-Aldrich (St.Louis, USA)	1:20000 in PBS containing 5% BSA

### Cytokine and chemokine determination

Concentrations of IL-6, chemokine (C-X-C) motif ligand 2 (CXCL-2) and chemokine (C-C motif) ligand 2 (CCL-2) were detected by commercial available ELISA kits (Thermo Scientific; Invitrogen, Darmstadt, Germany).

### Verification of cell density and formation of tight junctions

The appropriateness of L2 cell density to cover the silicon membrane, and development of tight junctions were evaluated in separate experiments. Briefly, AECs were seeded and stretched as described above. Those cells were washed with phosphate buffered saline (PBS), fixed with 4% (w/v) paraformaldehyde in PBS (12 min at room temperature/RT), permeabilized with ice cold 0.1% (v/v) Triton X-100 in PBS (5 min, RT) and then blocked with 3% (w/v) bovine serum albmin in PBS (20–30 min, RT). Cells were incubated with Phalloidin-Alexa (1:500), or labeled with the primary antibody against VE-cadherin for 1 hour at room temperature, then washed with PBS (2 x 3 min), then labeled with the secondary antibody Alexa-Fluor-488 goat anti-rabbit (1:500), 30 min at room temperature, and washed with PBS (2 times x 3 min). DAPI (1:200) was added to the phalloidin Alexa 488 or secondary antibody solution. Afterwards, membranes were cut and mounted on microscopy slides using MOWIOL (Calbiochem/Merck, Germany). Cells were imaged using an LSM 510 confocal microscope equipped with a 40x numerical aperture N.A. = 1.3 oil DIC objective (Carl Zeiss Microimaging, Germany at the Imaging Facility Medizinisch-Theoretisches Zentrum (MTZ), Dresden), or an LSM710 microscope equipped with a 63x, N.A. = 1.4 objective (Carl Zeiss Microimaging, Germany, at the Imaging Facility, Biotechnology Institute, TU Dresden). Z-stacks were acquired with a 1 μm interval between consecutive focal planes. Images were analyzed and processed using a quantitative image analysis tool, ImageJ (National Institutes of Health, USA), and Photoshop^®^ CS2 (Acrobat Adobe 1999–2005). The 3D renditions were obtained from 0.5 μm Z-sections using the ImageJ 3D Viewer plugin (volume view) (Rasband, W.S., ImageJ, U. S. National Institutes of Health, Bethesda, Maryland, USA, http://imagej.nih.gov/ij/, 1997–2016).

### Statistical analysis

Data are reported as mean±SD of at least 4 experiments. GraphPad Prism 6.0 (GraphPad Software Inc., San Diego, USA) was used for the analysis. Group comparisons were performed with one-way ANOVA followed by the Tukey post hoc test (GraphPad Software Inc., San Diego, USA). Differences were considered statistically significant if p < 0.05.

## Results

### Cell density and formation of tight junctions

As shown in S4 and S5 Figs, the L2 cell density used was appropriate to cover the silicon membrane, and those cells developed tight junctions.

### Cell viability

After a period of 4h, non-variable and variable cyclic stretch resulted in similar release of LDH by L2 and type I-like AECs irrespective of LPS stimulation, as compared to non-stretched cells (S6 Fig).

### mRNA transcription of pro-inflammatory cytokines

**Fig 1:**
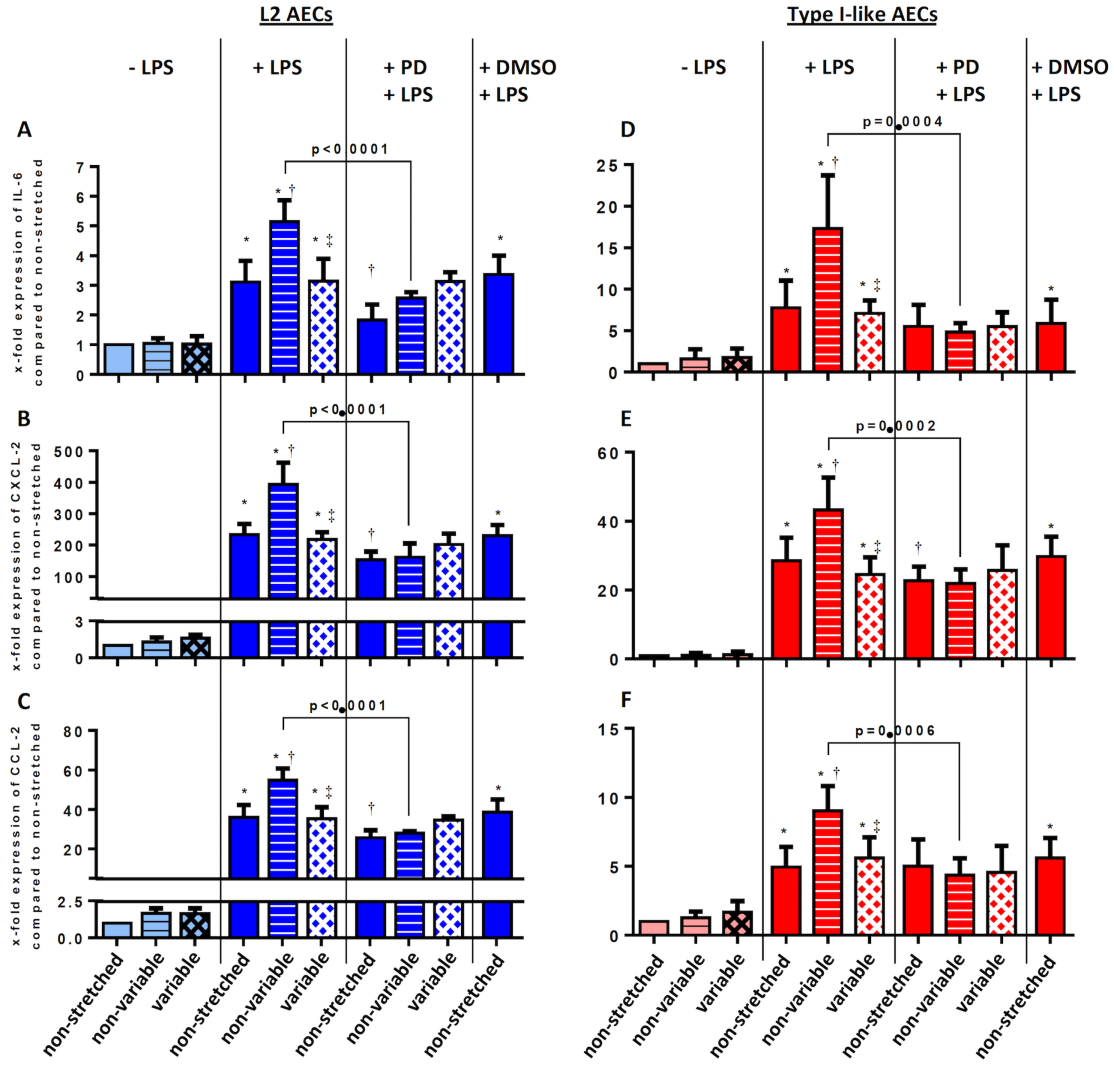
Effects of mechanical non-variable and variable stretch of L2 and type-I-like alveolar epithelial cells on gene expression of IL-6, CXCL2 and CCL2.

As shown in Fig 1, in L2 and type I-like AECs without LPS stimulation, variable and non-variable cyclic stretch did not increase the mRNA expression of IL-6, CXCL-2 and CCL-2. Stimulation with LPS significantly increased the mRNA expression of IL-6 (A, D), CXCL-2 (B, E) and CCL-2 (C, F), in non-stretched, as well as non-variable and variable stretched AECs. Non-variable, but not variable, cyclic stretch further increased the mRNA expression of these pro-inflammatory cytokines. Neither in L2 nor in type I-like AECs the mRNA expression of the anti-inflammatory cytokines IL-4 and IL-10 could be detected.

L2 (A, B, C) and type-I-like alveolar epithelial cells (D, E, F) were exposed to -/+ stretch, -/+ lipopolysaccharide (LPS, 2μg/ml), -/+ MEK/ERK1/2 Inhibitor IV (PD184161) and dimethyl sulfoxide (DMSO, vehicle control for PD 184161). RNA was isolated, reverse transcribed, and the cDNA products for (A, D) IL-6, (B, E) CXCL2 and (C, F) CCL2 were analyzed by semiquantitative RT-PCR using the ΔΔCT method. Data are normalized to non-stretched cells. Stretch was adjusted to the cells with a frequency of 0.5 Hz. Data are means ± standard deviation of at least 4 experiments. *p<0.05, relative to non-stretched, † p<0.05, relative to LPS+non-stretched; ‡ p<0.05, relative to LPS+non-variable stretch.

### Protein expression of pro-inflammatory cytokines

**Fig 2:**
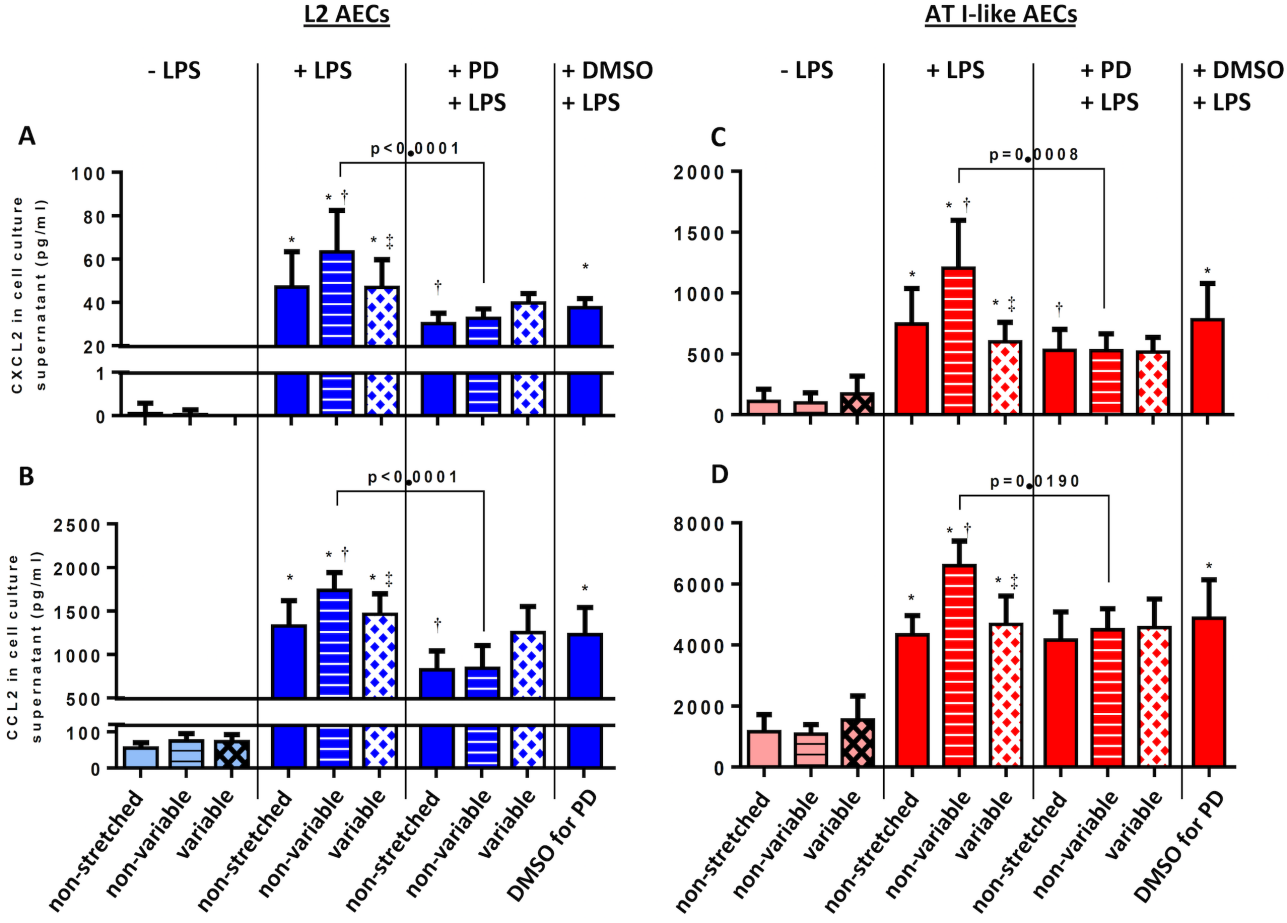
Effects of non-variable and variable mechanical stretch of L2 and type I-like alveolar epithelial cells on the release of CXCL2 and CCL2.

In L2 and type I-like AECs without LPS stimulation, neither non-variable, nor variable cyclic stretch increased the release of CXCL-2 and CCL-2, but LPS stimulation resulted in an increase of these cytokines irrespective of stretch type (Fig 2). Non-variable, but not variable cyclic stretch, further increased the release of CXCL-2 and CCL-2 compared to LPS challenge. In L2 AECs, the release of IL-6 presented the same behavior as CXCL-2 and CCL-2 (S7 Fig). In type I-like AECs, the IL-6 release was lower than the detection limit.

L2 (A, B) and type I-like alveolar epithelial cells (C, D) were exposed to -/+ stretch, -/+ lipopolysaccharide (LPS, 2μg/ml), /+ MEK/ERK1/2 Inhibitor IV (PD184161) and dimethyl sulfoxide (DMSO, vehicle control for PD 184161). Cell culture supernatants were analyzed for (A, C) CXCL2, (B, D) CCL2 by ELISA Kits. Stretch was adjusted to the cells with a frequency of 0.5 Hz. Data are means ± standard deviation of at least 4 experiments. *p<0.05, relative to non-stretched, † p<0.05, relative to LPS+non-stretched; ‡ p<0.05, relative to LPS+non-variable stretch, § p<0.05, relative to LPS+variable stretch.

### Signaling pathways

#### ERK1/2 and SAPK/JNK

**Fig 3:**
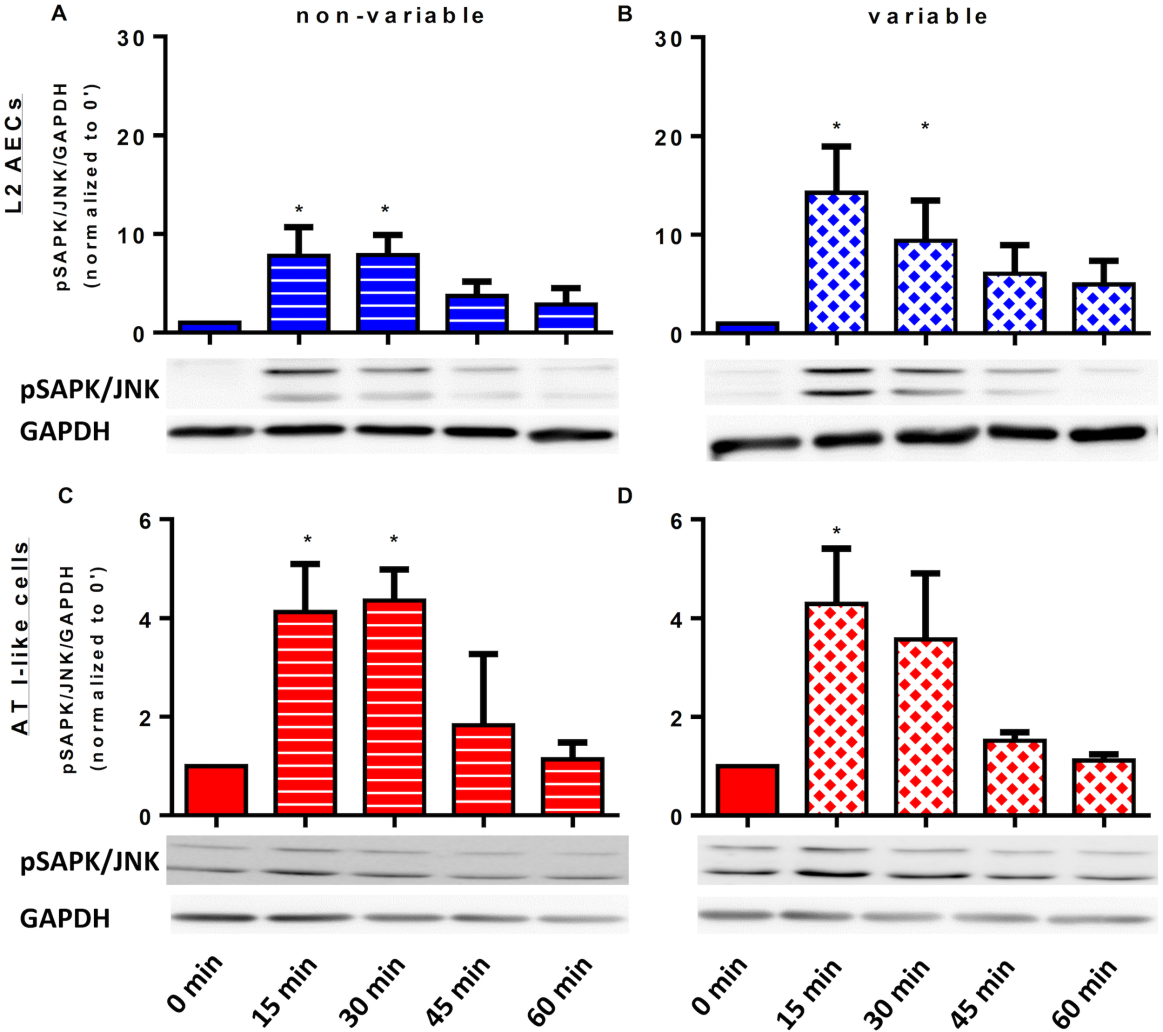
Time course of stress-activated protein kinase (SAPK) phosphorylation in homogenates of L2 and type-I-like alveolar epithelial cells.

Fig 3 shows representative immunoblots and respective histograms of activated SAPK/JNK normalized to GAPDH band intensity. Compared to non-stretched cells, SAPK/JNK phosphorylation increased significantly within 15 minutes after start of both non-variable and variable cyclic stretch after the stimulation by LPS in L2 and type I-like AECs. After 45 minutes, SAPK/JNK phosphorylation approximately returned to non-stretched levels.

L2 and type-I-like alveolar epithelial cells (AECs) were left non-stretched (time point 0), non-variable stretched (7.5%) or variable stretched (1–15%, SD 2.5%) for 15, 30, 45 and 60 min, with lipopolysaccharide (LPS, 2μg/ml) stimulation for 1h. Phosphorylated SAPK/JNK and GAPDH were analyzed by immunoblot using specific antibodies. Densitometric values are shown as fold increases over non-stretched (time point 0). (A) non-variable—L2 AECs, (B) variable L2 AECs, (C) non-variable AT I-like cells, (D) variable AT I-like cells. Data are means ± standard deviation of at least 4 experiments. *p<0.05 relative to non-stretched.

**Fig 4:**
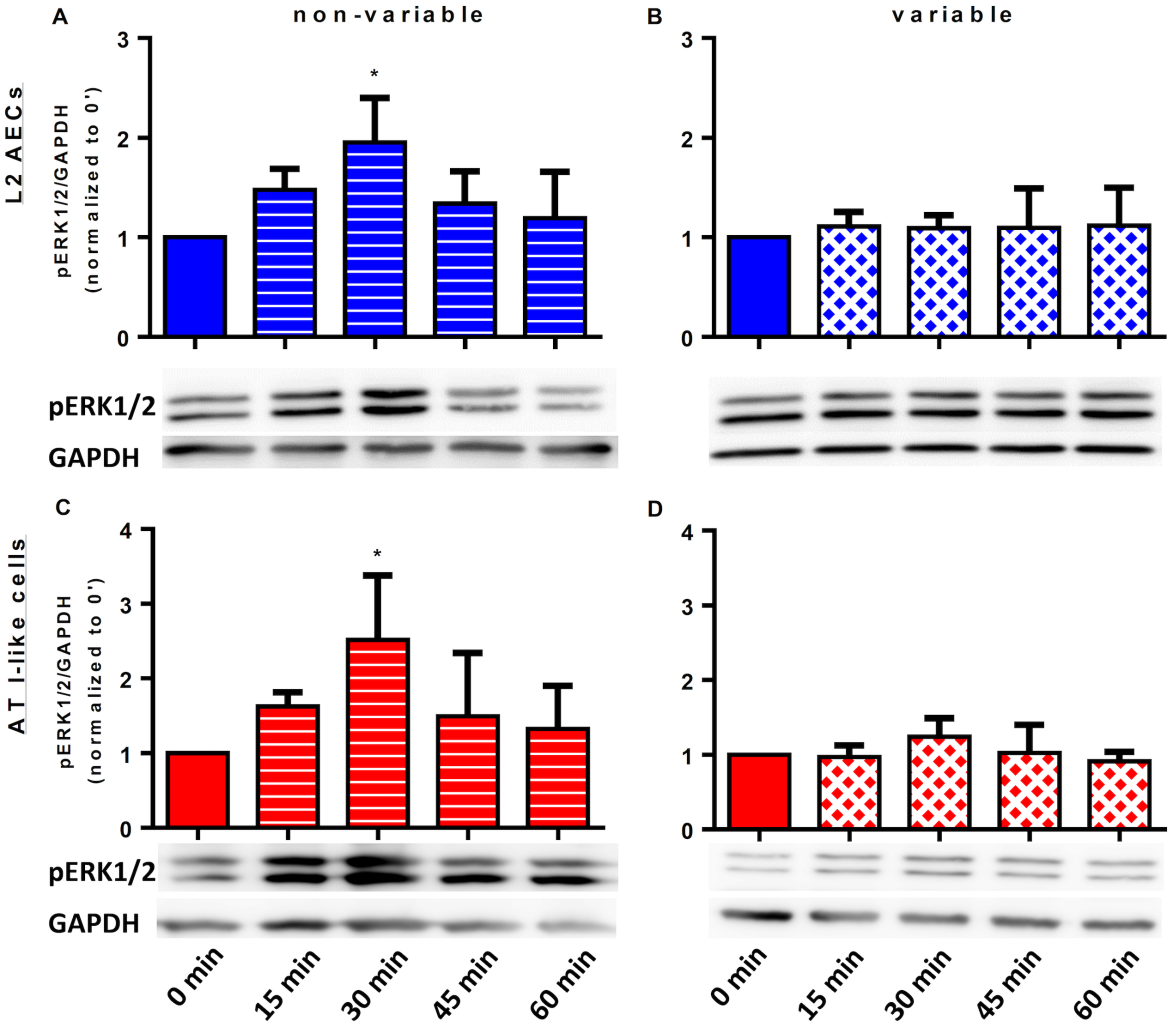
Time course of extracellular signal-regulated kinases (ERK)-1/2 phosphorylation in homogenates of L2 and type-I-like alveolar epithelial cells.

The behavior of ERK1/2 phosphorylation in LPS stimulated L2 and type I-like AECs is depicted in Fig 4. In both types of AECs, non-variable cyclic stretch induced a phosphorylation peak of ERK1/2 after 30 minutes, and values returned to approximately non-stretched levels after 60 minutes (Fig 4A and 4C, respectively). In contrast, variable stretched L2 and type I-like AECs cells did not show any significant changes in ERK1/2 phosphorylation after LPS stimulation (Fig 4B and 4D, respectively).

L2 and type I-like alveolar epithelial cells (AECs) were left non-stretched (control, time point 0), non-variable stretched (7.5%) or variable stretched (1–15%, SD 2.5%) for 15, 30, 45 and 60 min, with lipopolysaccharide (LPS, 2μg/ml) stimulation for 1h. Phosphorylated ERK1/2 and GAPDH were analyzed by immunoblot, using specific antibodies. Densitometric values are shown as fold increases over non-stretched (time point 0). (A) non-variable L2 AECs, (B) variable L2 AECs, (C) non-variable AT I-like cells, (D) variable AT I-like cells. Data are means ± standard deviation of at least 4 experiments. *p<0.05 relative to non-stretched.

#### Focal adhesion kinase

The phosphorylation of focal adhesion kinase (FAK) at Tyr^576^ was not influenced by LPS stimulation or cyclic stretch pattern in both types of AECs. An additional figure shows this in more detail (S8 Fig).

#### MEK/ERK1/2 inhibition

In LPS-stimulated L2 and type I-like AECs submitted to non-variable, but not variable cyclic stretch, the inhibition of ERK1/2 phosphorylation by the MEK inhibitor (PD184161) partially suppressed the expression and release of IL-6, CXCL-2 and CCL-2 (Figs 1 and 2, respectively).

#### SAPK/JNK inhibition

In non-stretched and stretched LPS-stimulated L2 AECs, SAPK/JNK inhibition reduced the expression and release of IL-6, CXCL-2 and CCL-2 compared to the DMSO vehicle control. The nature of the cyclic stretch pattern did not influence these effects (S9 Fig).

## Discussion

The main finding of the present study is that, in LPS-stimulated, but not in non LPS-stimulated L2 and type I-like AECs, 4 hours of variable cyclic cell stretch decreased the gene expression and release of IL-6, CXCL-2 and CCL-2 compared to non-variable cyclic stretch, and also non-stretched cells. Within the non-injurious range of variable stretch tested (≤ 15%), and differently from non-variable stretch, inhibition of the ERK1/2 signaling pathway did not result in further reduction of gene expression and release of IL-6, CXCL-2 and CCL-2. Furthermore, non-variable cyclic stretch of LPS-stimulated L2 and type I-like AECs resulted in increased phosphorylation of the MAPK ERK1/2 after 30 minutes, compared to variable cyclic stretch.

To our knowledge, this is the first study addressing the pro-inflammatory response of AECs to variable cyclic stretch. We opted for the use of L2 AECs, not only because they are relatively easy to cultivate, but mainly the fact that they express the AEC type I markers caveolin-1, receptor for advanced glycation end products (RAGE) as well as T1-alpha, do not show surfactant A, B, C and D gene expression (see S10 and S11 Figs), and thus seem well suited to investigate the behavior of type-I AECs. Furthermore, we performed a series of stretch experiments in type I-like AECs to confirm results in the immortalized L2 cell line. Variable stretch in the range of 1–15% was chosen for different reasons. First, we aimed at a mean stretch that is compatible with the stretch of AECs during protective mechanical ventilation [20]. Second, we wanted to reproduce a coefficient of variation of approximately 30%, in analogy to studies that investigated the impact of variable tidal volumes on lung inflammation in experimental ARDS [21]. A further feature is that we tested variable AEC stretch without and with LPS challenge in order to mimic mechanical cell stress as first and second hits, respectively. In addition, we aimed not at cell death, i.e. the most extreme consequence of cell challenge, but rather the pro-inflammatory response, which may play a major role in the cell response to mechanical stress. Finally, we used a dedicated, customer-made device that is able to accomplish random variable cyclic stretch of commercially available silicon membranes, thus reproducing the environment of variable mechanical ventilation.

It is well known that high tidal volumes during mechanical ventilation can initiate or worsen lung injury, a phenomenon known as ventilator-induced lung injury (VILI) [22]. Therefore, protective strategies based upon low distending pressures (driving pressure) and tidal volumes have been developed to reduce the stress of mechanical ventilation to the lung parenchyma [23]. In experimental ARDS, variable tidal volumes have been shown to improve respiratory lung function, and further reduce histological damage [2] as well as decrease cytokine release [1] compared to conventional (non-variable) protective low tidal volumes. However, the use of protective low tidal volumes may still cause lung damage both in patients with preexisting lung injury as well as in patients with healthy lungs [24]. Currently, it is not clear which specific lung cells trigger this phenomenon, but since type-I AECs cover more than 95% of the alveolar surface [25], they are likely importantly involved in the pathogenesis of VILI.

Our observation that cell death was practically negligible evidences that neither a stretch between 1–15%, nor the amount of LPS used for stimulation caused severe damage to AECs, allowing the assessment of the pro-inflammatory response within the cell viability range. Previous studies demonstrated that cyclic strain of more than 25% results in plasma membrane rupture and lead to cell death in type-II AECs of rats. Interestingly, moderate stretch of 15% has no negative influence to cell viability [26, 27], which is in agreement with our results. It is worth of note that the pro-inflammatory response of non LPS-stimulated L2 AECs was not increased at the levels of stretch used, suggesting that healthy AECs submitted to non-excessive mechanical strain do not contribute to the pro-inflammatory response of lungs.

The increased gene expression and release of IL-6, CXCL-2 and CCL-2 by LPS-stimulated L2 and type I-like AECs is in agreement with previous reports showing that LPS is a potent trigger of the pro-inflammatory response in AECs [28, 29]. Also, our observation that the pro-inflammatory response of LPS-stimulated AECs was further increased by non-variable cyclic stretch is in line with the so-called “two-hit hypothesis” of VILI [30]. In experimental ARDS in rats, LPS stimulation and mechanical ventilation have been shown to act synergistically, amplifying the release of IL-6, IL-1β, TNF-α and MIP-2 (CXCL-2) in the bronchoalveolar lavage and this cellular response is mediated by activation of MAPKs, e.g. ERK1/2, SAPK/JNK and p38 [31]. It has been shown that LPS-stimulation and mechanical ventilation, as well as combination of these challenges, lead to phosphorylation of MAPKs in rat lungs [31]. According to the present data, SAPK/JNK and ERK1/2 achieve maximal phosphorylation approximately 15 to 30 minutes of non-variable cyclic stretch, independently of the stimulation by LPS. Notably, the release of pro-inflammatory cytokines by L2 and type I-like AECs was lower during variable than non-variable cyclic stretch, and comparable with the non-stretched control, suggesting that a variable stretching pattern obviated mechanical stress as a second hit in these cells.

The finding that the inhibition of SAPK/JNK and ERK1/2 reduced the release of pro-inflammatory cytokines in both non-variable stretched and non-stretched AECs, suggests that neither SAPK/JNK, nor ERK1/2 were involved in the cytokine release following stimulation by LPS. Accordingly, these pathways were also not involved in the pro-inflammatory response induced by non-variable cyclic stretch. Our results are, at least partly, in contrast with a previous investigation showing that cyclic strain of 12% did not activate SAPK/JNK in primary type-II AECs (ATII) [16]. A possible explanation for these differences could be the use of distinct cell types, namely ATII and ATI-like cells. The cell line L2 has been originally obtained from adult rats and depicts type-II-like properties in an early phase of culture, for example lamellar bodies, but those properties change across time of culture [32], yielding type-I-like cells.

It has been shown that focal adhesion kinase (FAK) is activated by non-variable stretching in human epithelial cell lines [33]. In our study, however, phosphorylation of FAK was not influenced by LPS challenge or cell stretch pattern, suggesting that this protein kinase did not respond for the anti-inflammatory cellular mechanisms of variable stretch. In contrast, in LPS-stimulated AECs submitted to non-variable stretch, the inhibition of ERK1/2 decreased both the gene expression and release of IL-6, CXCL-2 and CCL-2, evidencing that this pathway was likely involved in the pro-inflammatory response. Accordingly, our observation that, during variable stretch in LPS-stimulated AECs, the inflammatory response was already comparable with non-stretched AECs and inhibition of the same pathway did not influence the release of cytokines indicates that ERK1/2 is a key pathway for the anti-inflammatory mechanism of variable cell stretch. Given that SAPK did not show a similar behavior as ERK1/2, i.e. its inhibition affected the pro-inflammatory response of AECs during both variable and non-variable stretch, this pathway is likely not involved in the cellular mechanisms of variable ventilation.

### Potential translational implications of the findings

The fact that a variable cyclic stretch pattern is able to reduce the pro-inflammatory response of AECs might not only have implications for the understanding of the mechanisms of variable ventilation, but could also contribute to explain, at least in part, the reduction of lung inflammation during mechanical ventilations modes that are associated with increased breath-by-breath variation of tidal volume and respiratory rate, for example pressure support ventilation [34], proportional assisted ventilation [35], biphasic positive airway pressure/airway pressure release ventilation [36], and neurally adjusted ventilatory assist [37]. Furthermore, our results suggest that the cellular mechanisms of VILI, as well as pharmacological intervention possibilities, might differ in presence of variability in the cell stretch pattern. Clearly, the present findings indicate that further research on the molecular basis of VILI under conditions of variable mechanical stress is warranted.

### Limitations

Our study knows several limitations. First, the results were obtained in AECs that show type-I characteristics, and extrapolation to type-II AECs may not be appropriate. Second, cells were obtained from rats, precluding direct extrapolation to other species. Third, the *in vitro* conditions where AECs grew, including lack of cell-to-air or cell-surfactant layers, do not reproduce the much more complex environment of the lung parenchyma. Nevertheless, when using this setting we were able to exclude potential confounders of whole animal models, for example decreased mechanical stress resulting from lung recruitment, which may also reduce the inflammatory response. Fourth, we investigated a few selected pathways of cell response to variable cyclic mechanical stress. Fifth, we challenged cells with a particular LPS compound as a first hit, which may contain also bacterial DNA, lipo-proteins and other constituents. However, this study was conceived as a proof-of-concept, and the use of other challenge compounds as well as the assessment of further pathways, which might also be involved in the response to variable stretch, were beyond its scope. Sixth, although the amplitude of cell stretch has been derived from previous studies in the literature, the exact amount of AEC strain is not well determined.

## Conclusion

In LPS-stimulated L2 and type I-like ACEs, variable compared to non-variable cyclic stretch decreased the gene activation and release of pro-inflammatory cytokines. These effects were potentially mediated by ERK1/2 pathway of cell response to mechanical stress.

## Supporting information

S1 Fig Expression of alveolar type I (caveolin-1 and aquaporin-5) and II markers (pro-surfactant protein-D) in primary alveolar epithelial cells after 5 days of culture in our laboratory. Measurements were obtained by immunoblots using specific antibodies. A protein extract from rat lung tissue served as positive control.
(DOCX)

S2 Fig Device for stretching of alveolar epithelial cells. Three cylindrical intenders (1) were used to apply homogeneous stretch on the silicon membranes of a BioFlex culture plate (2). A brushless motor (3) drives the hosting gear (4) to perform the vertical displacement of the intenders.
(DOCX)

S3 Fig Patterns of tidal stretch of alveolar epithelial cells that grow on a silicon membrane. (A) non-variable cell stretching pattern (7.5%); (B) variable cell stretching pattern (random variable peak between 1 and 15%, mean peak of 7.5%, normal distribution). White line: strain amplitude of 7.5%.
(DOCX)

S4 Fig AECs from L2 cell line were either non-stretched or stretched during periods of 1h and 4h. Cells were stained with Alexa 488 Phalloidin antibody (actin filaments); DNA was stained by DAPI and data were recorded using a confocal microscopy with an objective 40x and 60x. Data are displayed as a projection from 0.5 μm Z-sections. Single channels are in gray scale for DAPI and Phallodin; Merge: Phalloidin (green), DAPI (blue). (A) non-stretched, (B) stretched 1h, (C) stretched 4h in 40x objective and (D) non-stretched, (E) stretched 1h and (F) stretched 4h in a 60x objective. The Scale bar: 0.5 μm.
(DOCX)

S5 Fig AECs from L2 cell line were non-stretched and stretched during 1h and 4h. AECs were stretched and stained with the anti-rabbit VE-cadherin antibody, mediate the intercellular junction; DNA was stained by DAPI and data were recorded using a confocal microscopy with a 60x objective. The arrows are showing the tight junctions between cells. Data are displayed as a projection from 0.5 μm Z-sections stacks. Single channels are in gray scale for DAPI and VE-cadherin, indicated at the top; Merge: VE-cadherin (green), DAPI (blue). (A) non-stretched, (B) stretched 1h, (C) stretched 4h. Scale bar: 0.5 μm.
(DOCX)

S6 Fig Lactate dehydrogenase (LDH) activity in supernatants of rat L2 and type-I-like alveolar epithelial cells. L2 (A) and alveolar type-I-like epithelial cells (B) were exposed to cyclic non-variable or variable stretch for 4 hours with and without lipopolysaccharide stimulation (2μg/ml). Stretch was adjusted to the cells with a frequency of 0.5 Hz. Data are means ± standard deviation of at least four experiments performed in duplets.
(DOCX)

S7 Fig Effect of mechanical stretch on the release of interleukin(IL)-6 by L2 alveolar epithelial cells (AECs). L2 were exposed to -/+ stretch, -/+ lipopolysaccharide (LPS, 2μg/ml), /+ MEK/ERK1/2 Inhibitor IV (PD184161) and dimethyl sulfoxide (DMSO, vehicle control for PD 184161). Cell culture supernatants were analyzed for IL-6 by an ELISA Kit. Stretch was adjusted to the cells with a frequency of 0.5 Hz. Data are means ± standard deviation of at least 4 experiments. *p<0.05, relative to non-stretched, † p<0.05, relative to LPS+non-stretched; ‡ p<0.05, relative to LPS+non-variable stretch, § p<0.05, relative to LPS+variable stretch.
(DOCX)

S8 Fig Time course of focal adhesion kinase (FAK) phosphorylation in L2 and primary type-I-like alveolar epithelial cell (AEC) homogenates. L2 and type-I-like AECs were left non-stretched (time point 0), non-variable stretched (7.5%) or variable stretched (1–15%, SD 2.5%) for 15, 30, 45 and 60 min, with LPS (2μg/ml) priming for 1h. Phosphorylated FAK at Tyr^576^ and GAPDH were analyzed by immunoblot, using specific antibodies. Densitometric values are shown as fold increases over non-stretched cells. (A) non-variable L2 AECs, (B) variable L2 AECs, (C) non-variable primary AT I-like AECs, (D) variable primary AT I-like AECs. Data are means ± standard deviation of at least 4 experiments.
(DOCX)

S9 Fig Effects of mechanical non-variable and variable stretch of L2 alveolar epithelial cells on gene expression and release of IL-6, CXCL2 and CCL2. L2 alveolar epithelial cells were exposed to -/+ stretch, -/+ lipopolysaccharide (LPS, 2μg/ml), -/+ JNK inhibitor II (SP600125) and dimethyl sulfoxide (DMSO, vehicle control for SP 600125). RNA was isolated, reverse transcribed, and the cDNA products for (A) IL-6, (B) CXCL2 and (C) CCL2 were analyzed by semiquantitative RT-PCR using the ΔΔCT method. Data are normalized to non-stretched cells. Cell culture supernatants were analyzed for (D) IL-6, (E) CXCL-2, (F) CCL-2 by ELISA Kits. Stretch was adjusted to the cells with a frequency of 0.5 Hz. Data are means ± standard deviation of at least 4 experiments. *p<0.05, relative to non-stretched, † p<0.05, relative to LPS+non-stretched; ‡ p<0.05, relative to LPS+non-variable stretch; § p<0.05, relative to LPS+variable stretch.
(DOCX)

S10 Fig Expression of alveolar type (AT) I markers in L2 alveolar epithelial cells. RNA was isolated, reverse transcribed and the PCR products of the cDNA were separated by gel electrophoresis. DNA fragments were synthesized by PCR with primers for the AT I specific genes caveolin-1 (*lane 1*), receptor for advanced glycation end products (*lane 2*) and T1-alpha (*lane 3*). *Lane 4* shows DNA fragment molecular weight standard.
(DOCX)

S11 Fig Expression of alveolar type (AT) II markers in L2 alveolar epithelial cells. RNA was isolated, reverse transcribed and the PCR products of the cDNA were separated by gel electrophoresis. DNA fragments were synthesized by PCR with primers for the AT II specific genes surfactant protein-A (*lane 1*), surfactant protein-B (*lane 2*), surfactant protein-C (*lane 3*) and surfactant protein-D (*lane 4*). *Lane 5* shows DNA fragment molecular weight standard. As positive control served cDNA of rat lung tissue.
(DOCX)
