# Supplementary material for: Variable stretch reduces the pro-inflammatory response of alveolar epithelial cells
Source: PLoS One. 2017 Aug 15;12(8):e0182369. doi: 10.1371/journal.pone.0182369 (PMC5557541; doi:10.1371/journal.pone.0182369)
Supplement: S1 Fig — Measurements were obtained by immunoblots using specific antibodies. A protein extract from rat lung tissue served as positive control. (DOCX) [file pone.0182369.s001.docx]

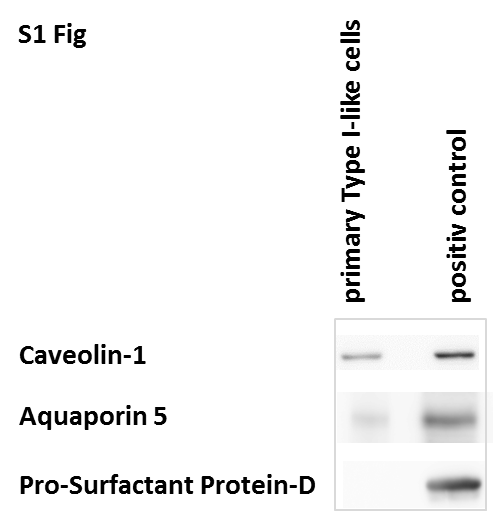


**S1 Fig - Expression of alveolar type I (caveolin-1 and aquaporin-5) and II markers (pro-surfactant protein-D) in primary alveolar epithelial cells after 5 days of culture in our laboratory**. Measurements were obtained by immunoblots using specific antibodies. A protein extract from rat lung tissue served as positive control.
