# Supplementary material for: Variable stretch reduces the pro-inflammatory response of alveolar epithelial cells
Source: PLoS One. 2017 Aug 15;12(8):e0182369. doi: 10.1371/journal.pone.0182369 (PMC5557541; doi:10.1371/journal.pone.0182369)
Supplement: S2 Fig — Three cylindrical intenders (1) were used to apply homogeneous stretch on the silicon membranes of a BioFlex culture plate (2). A brushless motor (3) drives the hosting gear (4) to perform the vertical displacement of the intenders. (DOCX) [file pone.0182369.s002.docx]

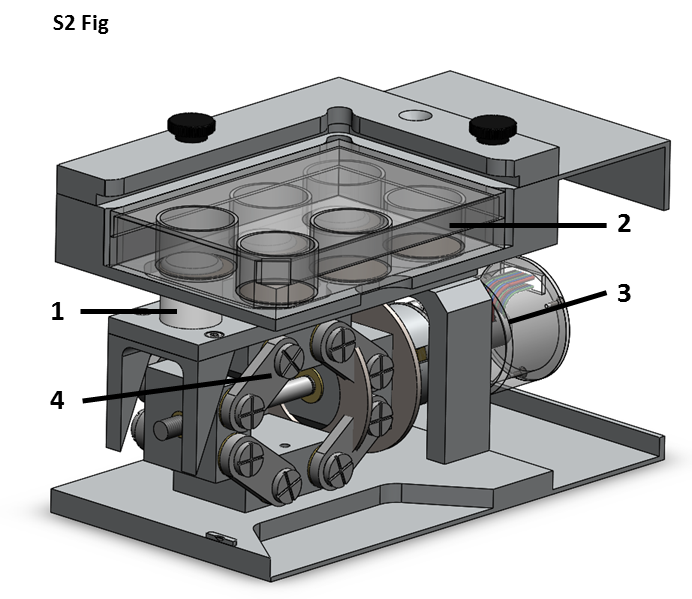


**S2 Fig - Device for stretching of alveolar epithelial cells.** Three cylindrical intenders (1) were used to apply homogeneous stretch on the silicon membranes of a BioFlex culture plate (2). A brushless motor (3) drives the hosting gear (4) to perform the vertical displacement of the intenders.
