# Supplementary material for: Variable stretch reduces the pro-inflammatory response of alveolar epithelial cells
Source: PLoS One. 2017 Aug 15;12(8):e0182369. doi: 10.1371/journal.pone.0182369 (PMC5557541; doi:10.1371/journal.pone.0182369)
Supplement: S3 Fig — (A) non-variable cell stretching pattern (7.5%); (B) variable cell stretching pattern (random variable peak between 1 and 15%, mean peak of 7.5%, normal distribution). White line: strain amplitude of 7.5%. (DOCX) [file pone.0182369.s003.docx]

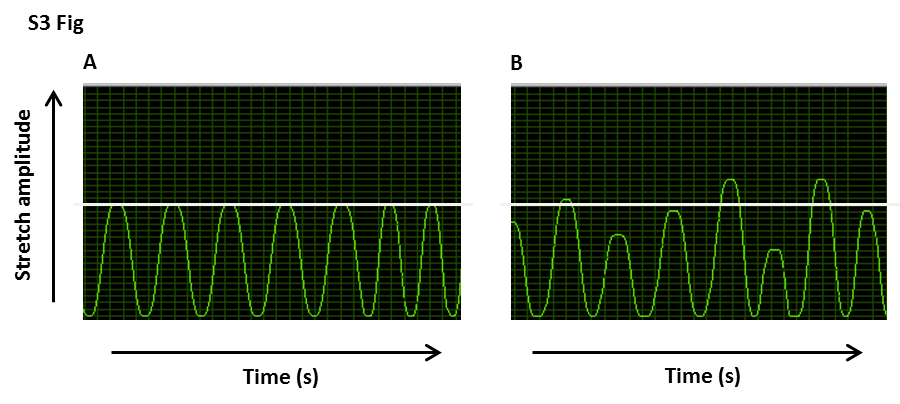


**S3 Fig - Patterns of tidal stretch of alveolar epithelial cells that grow on a silicon membrane.** (A) non-variable cell stretching pattern (7.5%); (B) variable cell stretching pattern (random variable peak between 1 and 15%, mean peak of 7.5%, normal distribution). White line: strain amplitude of 7.5%.
