# Supplementary material for: Variable stretch reduces the pro-inflammatory response of alveolar epithelial cells
Source: PLoS One. 2017 Aug 15;12(8):e0182369. doi: 10.1371/journal.pone.0182369 (PMC5557541; doi:10.1371/journal.pone.0182369)
Supplement: S4 Fig — Cells were stained with Alexa 488 Phalloidin antibody (actin filaments); DNA was stained by DAPI and data were recorded using a confocal microscopy with an objective 40x and 60x. Data are displayed as a projection from 0.5 μm Z-sections. Single channels are in gray scale for DAPI and Phallodin; Merge: Phalloidin (green), DAPI (blue). (A) non-stretched, (B) stretched 1h, (C) stretched 4h in 40x objective and (D) non-stretched, (E) stretched 1h and (F) stretched 4h in a 60x objective. The Scale bar: 0.5 μm. (DOCX) [file pone.0182369.s004.docx]

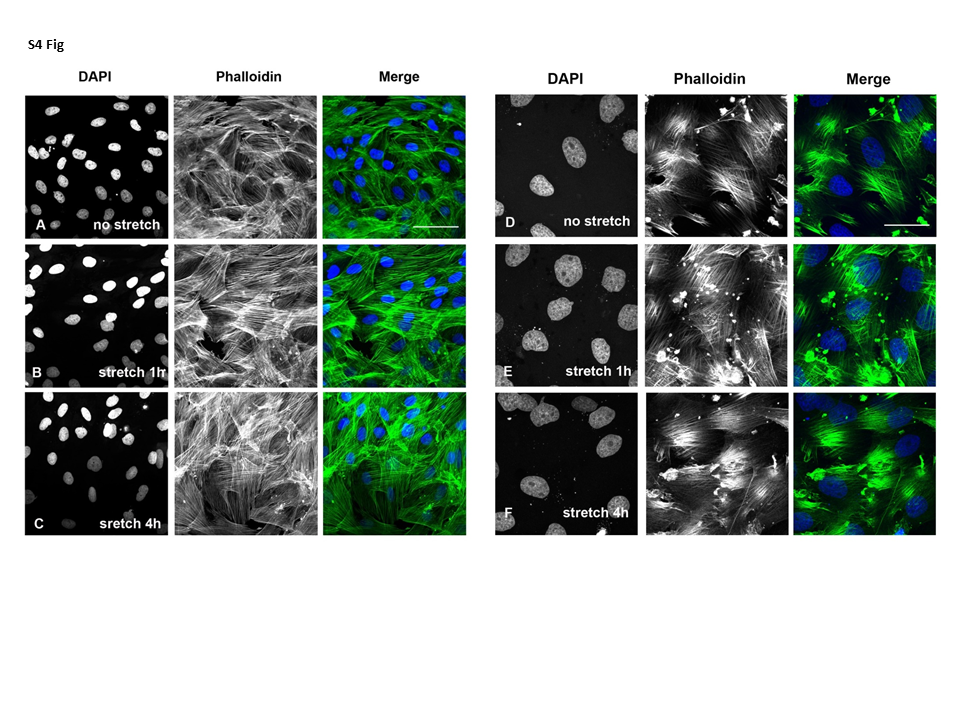


**S4 Fig -** **AECs from L2 cell line were either non-stretched or stretched during periods of 1h and 4h.** Cells were stained with Alexa 488 Phalloidin antibody (actin filaments); DNA was stained by DAPI and data were recorded using a confocal microscopy with an objective 40x and 60x. Data are displayed as a projection from 0.5 µm Z-sections. Single channels are in gray scale for DAPI and Phallodin; Merge: Phalloidin (green), DAPI (blue). (A) non-stretched, (B) stretched 1h, (C) stretched 4h in 40x objective and (D) non-stretched, (E) stretched 1h and (F) stretched 4h in a 60x objective. The Scale bar: 0.5 μm.
