# Supplementary material for: Variable stretch reduces the pro-inflammatory response of alveolar epithelial cells
Source: PLoS One. 2017 Aug 15;12(8):e0182369. doi: 10.1371/journal.pone.0182369 (PMC5557541; doi:10.1371/journal.pone.0182369)
Supplement: S5 Fig — AECs were stretched and stained with the anti-rabbit VE-cadherin antibody, mediate the intercellular junction; DNA was stained by DAPI and data were recorded using a confocal microscopy with a 60x objective. The arrows are showing the tight junctions between cells. Data are displayed as a projection from 0.5 μm Z-sections stacks. Single channels are in gray scale for DAPI and VE-cadherin, indicated at the top; Merge: VE-cadherin (green), DAPI (blue). (A) non-stretched, (B) stretched 1h, (C) stretched 4h. Scale bar: 0.5 μm. (DOCX) [file pone.0182369.s005.docx]

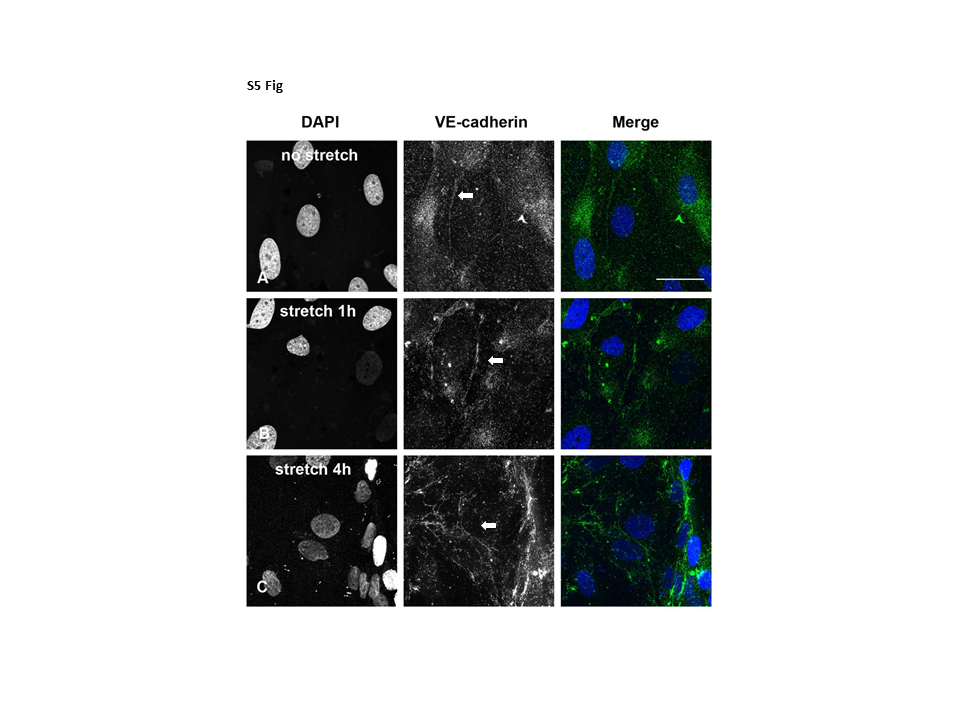


**S5 Fig - AECs from L2 cell line were non-stretched and stretched during 1h and 4h.** AECs were stretched and stained with the anti-rabbit VE-cadherin antibody, mediate the intercellular junction; DNA was stained by DAPI and data were recorded using a confocal microscopy with a 60x objective. The arrows are showing the tight junctions between cells. Data are displayed as a projection from 0.5 µm Z-sections stacks. Single channels are in gray scale for DAPI and VE-cadherin, indicated at the top; Merge: VE-cadherin (green), DAPI (blue). (A) non-stretched, (B) stretched 1h, (C) stretched 4h. Scale bar: 0.5 μm.
