# Supplementary material for: Variable stretch reduces the pro-inflammatory response of alveolar epithelial cells
Source: PLoS One. 2017 Aug 15;12(8):e0182369. doi: 10.1371/journal.pone.0182369 (PMC5557541; doi:10.1371/journal.pone.0182369)
Supplement: S6 Fig — L2 (A) and alveolar type-I-like epithelial cells (B) were exposed to cyclic non-variable or variable stretch for 4 hours with and without lipopolysaccharide stimulation (2μg/ml). Stretch was adjusted to the cells with a frequency of 0.5 Hz. Data are means ± standard deviation of at least four experiments performed in duplets. (DOCX) [file pone.0182369.s006.docx]

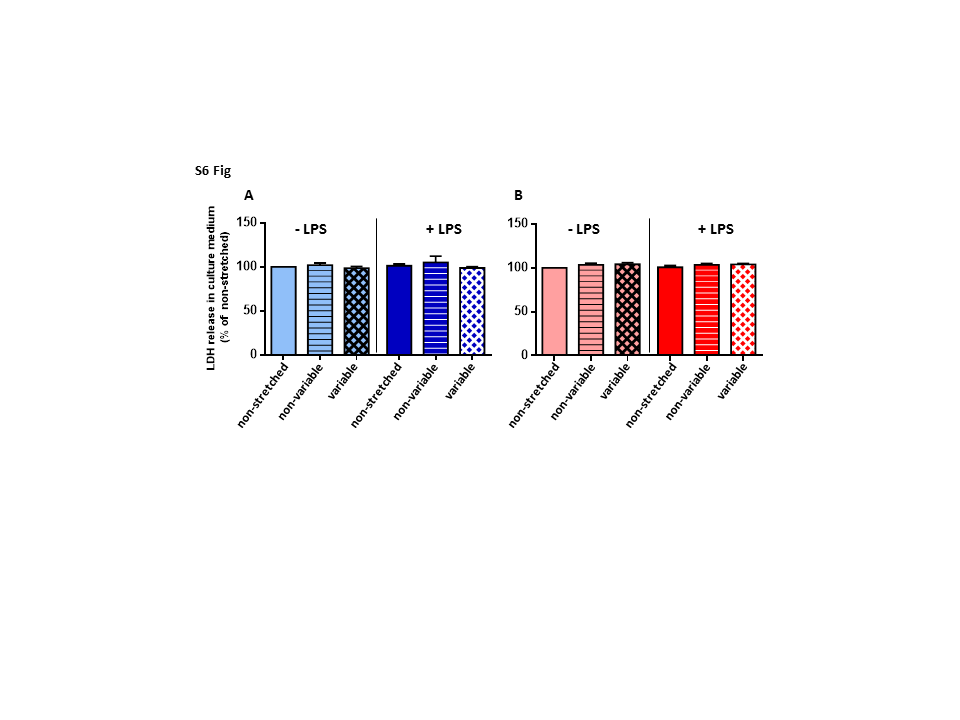


**S6 Fig -Lactate dehydrogenase (LDH) activity in supernatants of rat L2 and type-I-like alveolar epithelial cells.**

L2 (A) and alveolar type-I-like epithelial cells (B) were exposed to cyclic non-variable or variable stretch for 4 hours with and without lipopolysaccharide stimulation (2µg/ml). Stretch was adjusted to the cells with a frequency of 0.5 Hz. Data are means ± standard deviation of at least four experiments performed in duplets.
