# Supplementary material for: Variable stretch reduces the pro-inflammatory response of alveolar epithelial cells
Source: PLoS One. 2017 Aug 15;12(8):e0182369. doi: 10.1371/journal.pone.0182369 (PMC5557541; doi:10.1371/journal.pone.0182369)
Supplement: S7 Fig — L2 were exposed to -/+ stretch, -/+ lipopolysaccharide (LPS, 2μg/ml), /+ MEK/ERK1/2 Inhibitor IV (PD184161) and dimethyl sulfoxide (DMSO, vehicle control for PD 184161). Cell culture supernatants were analyzed for IL-6 by an ELISA Kit. Stretch was adjusted to the cells with a frequency of 0.5 Hz. Data are means ± standard deviation of at least 4 experiments. *p<0.05, relative to non-stretched, † p<0.05, relative to LPS+non-stretched; ‡ p<0.05, relative to LPS+non-variable stretch, § p<0.05, relative to LPS+variable stretch. (DOCX) [file pone.0182369.s007.docx]

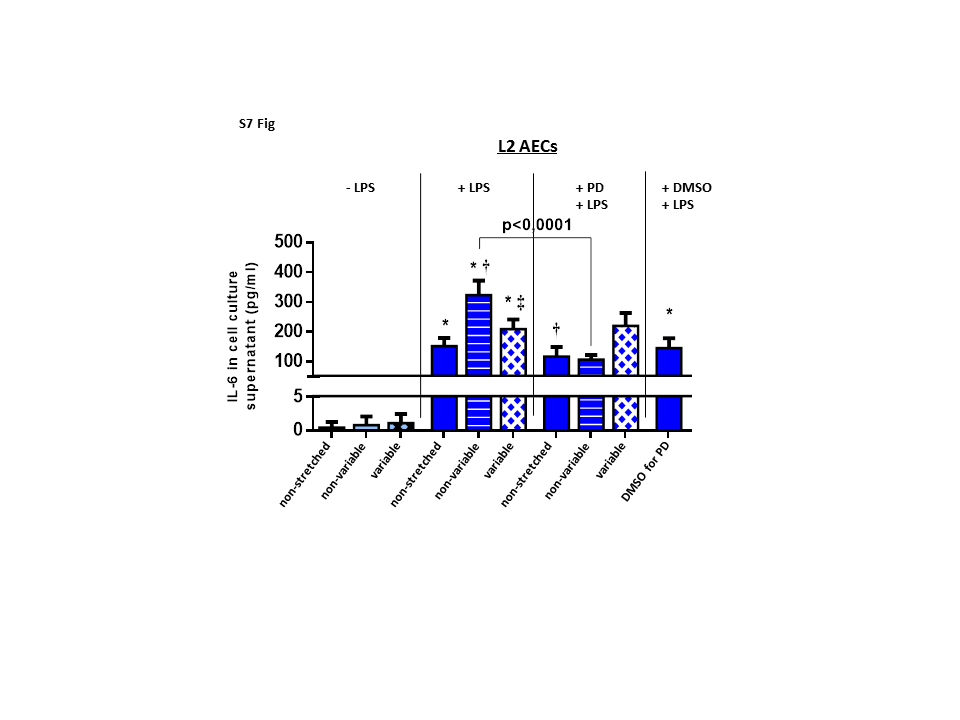


**S7 Fig - Effect of mechanical stretch on the release of interleukin(IL)-6 by L2 alveolar epithelial cells (AECs)**.

L2 were exposed to -/+ stretch, -/+ lipopolysaccharide (LPS, 2µg/ml), /+ MEK/ERK1/2 Inhibitor IV (PD184161) and dimethyl sulfoxide (DMSO, vehicle control for PD 184161). Cell culture supernatants were analyzed for IL-6 by an ELISA Kit. Stretch was adjusted to the cells with a frequency of 0.5 Hz. Data are means ± standard deviation of at least 4 experiments. *p<0.05, relative to non-stretched, † p<0.05, relative to LPS+non-stretched; ‡ p<0.05, relative to LPS+non-variable stretch, § p<0.05, relative to LPS+variable stretch.
