# Supplementary material for: Variable stretch reduces the pro-inflammatory response of alveolar epithelial cells
Source: PLoS One. 2017 Aug 15;12(8):e0182369. doi: 10.1371/journal.pone.0182369 (PMC5557541; doi:10.1371/journal.pone.0182369)
Supplement: S8 Fig — L2 and type-I-like AECs were left non-stretched (time point 0), non-variable stretched (7.5%) or variable stretched (1–15%, SD 2.5%) for 15, 30, 45 and 60 min, with LPS (2μg/ml) priming for 1h. Phosphorylated FAK at Tyr576 and GAPDH were analyzed by immunoblot, using specific antibodies. Densitometric values are shown as fold increases over non-stretched cells. (A) non-variable L2 AECs, (B) variable L2 AECs, (C) non-variable primary AT I-like AECs, (D) variable primary AT I-like AECs. Data are means ± standard deviation of at least 4 experiments. (DOCX) [file pone.0182369.s008.docx]

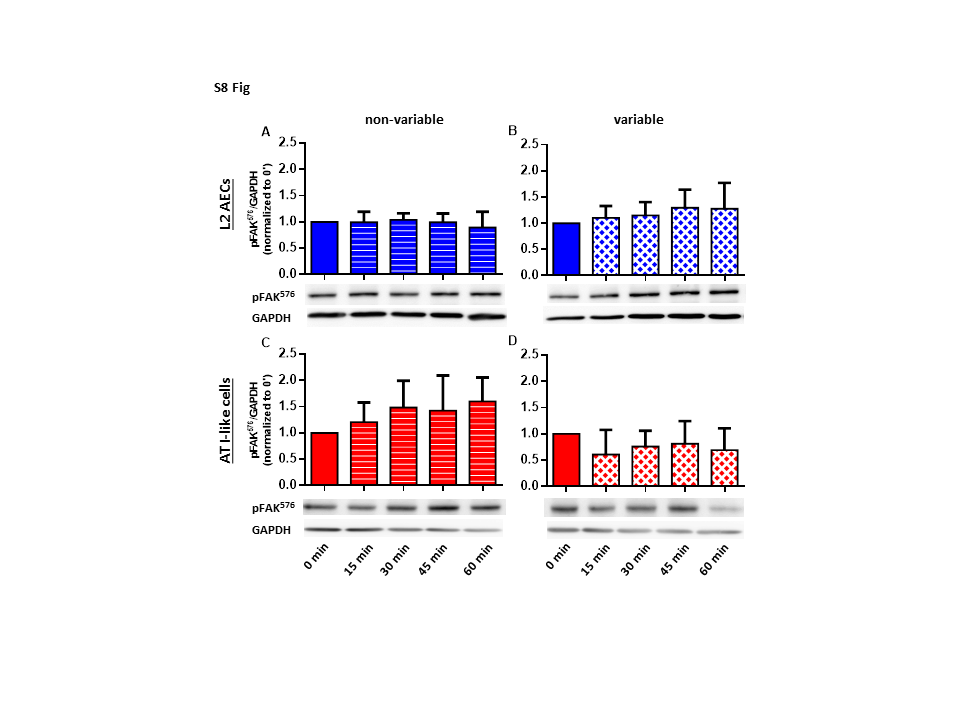


**S8 Fig -** **Time course of focal adhesion kinase (FAK) phosphorylation in L2 and primary type-I-like alveolar epithelial cell (AEC) homogenates**.

L2 and type-I-like AECs were left non-stretched (time point 0), non-variable stretched (7.5%) or variable stretched (1-15%, SD 2.5%) for 15, 30, 45 and 60 min, with LPS (2µg/ml) priming for 1h. Phosphorylated FAK at Tyr^576^ and GAPDH were analyzed by immunoblot, using specific antibodies. Densitometric values are shown as fold increases over non-stretched cells. (A) non-variable L2 AECs, (B) variable L2 AECs, (C) non-variable primary AT I-like AECs, (D) variable primary AT I-like AECs. Data are means ± standard deviation of at least 4 experiments.
