# Supplementary material for: Variable stretch reduces the pro-inflammatory response of alveolar epithelial cells
Source: PLoS One. 2017 Aug 15;12(8):e0182369. doi: 10.1371/journal.pone.0182369 (PMC5557541; doi:10.1371/journal.pone.0182369)
Supplement: S9 Fig — L2 alveolar epithelial cells were exposed to -/+ stretch, -/+ lipopolysaccharide (LPS, 2μg/ml), -/+ JNK inhibitor II (SP600125) and dimethyl sulfoxide (DMSO, vehicle control for SP 600125). RNA was isolated, reverse transcribed, and the cDNA products for (A) IL-6, (B) CXCL2 and (C) CCL2 were analyzed by semiquantitative RT-PCR using the ΔΔCT method. Data are normalized to non-stretched cells. Cell culture supernatants were analyzed for (D) IL-6, (E) CXCL-2, (F) CCL-2 by ELISA Kits. Stretch was adjusted to the cells with a frequency of 0.5 Hz. Data are means ± standard deviation of at least 4 experiments. *p<0.05, relative to non-stretched, † p<0.05, relative to LPS+non-stretched; ‡ p<0.05, relative to LPS+non-variable stretch; § p<0.05, relative to LPS+variable stretch. (DOCX) [file pone.0182369.s009.docx]

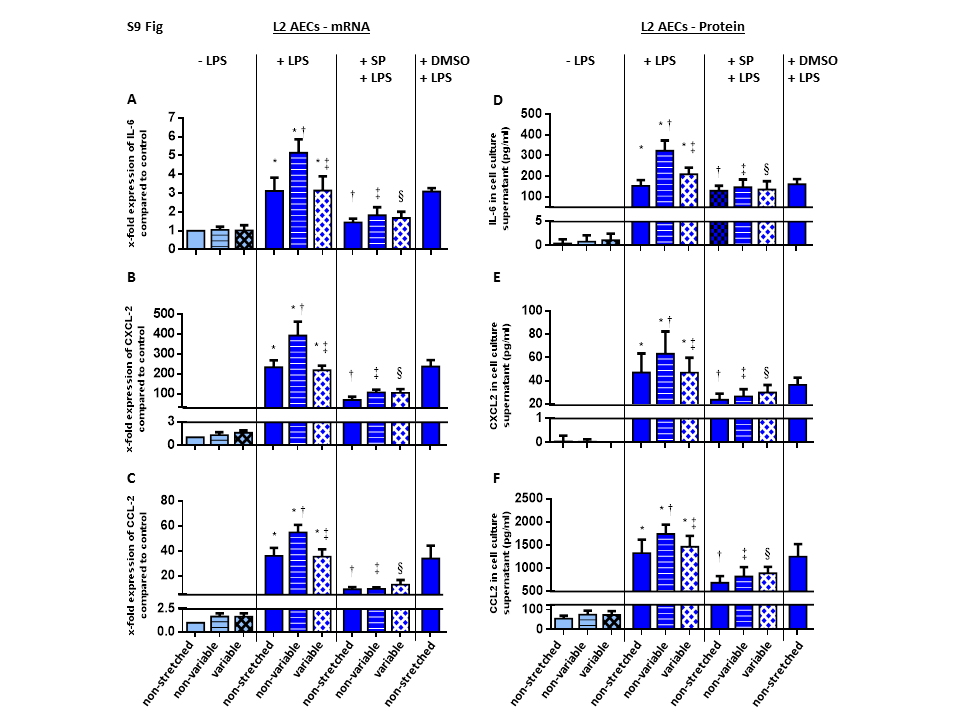


**S9 Fig - Effects of mechanical non-variable and variable stretch of L2 alveolar epithelial cells on gene expression and release of IL-6, CXCL2 and CCL2.**

L2 alveolar epithelial cells were exposed to -/+ stretch, -/+ lipopolysaccharide (LPS, 2µg/ml), -/+ JNK inhibitor II (SP600125) and dimethyl sulfoxide (DMSO, vehicle control for SP 600125). RNA was isolated, reverse transcribed, and the cDNA products for (A) IL-6, (B) CXCL2 and (C) CCL2 were analyzed by semiquantitative RT-PCR using the ∆∆CT method. Data are normalized to non-stretched cells. Cell culture supernatants were analyzed for (D) IL-6, (E) CXCL-2, (F) CCL-2 by ELISA Kits. Stretch was adjusted to the cells with a frequency of 0.5 Hz. Data are means ± standard deviation of at least 4 experiments. *p<0.05, relative to non-stretched, † p<0.05, relative to LPS+non-stretched; ‡ p<0.05, relative to LPS+non-variable stretch; § p<0.05, relative to LPS+variable stretch.
