# Supplementary material for: Variable stretch reduces the pro-inflammatory response of alveolar epithelial cells
Source: PLoS One. 2017 Aug 15;12(8):e0182369. doi: 10.1371/journal.pone.0182369 (PMC5557541; doi:10.1371/journal.pone.0182369)
Supplement: S10 Fig — RNA was isolated, reverse transcribed and the PCR products of the cDNA were separated by gel electrophoresis. DNA fragments were synthesized by PCR with primers for the AT I specific genes caveolin-1 (lane 1), receptor for advanced glycation end products (lane 2) and T1-alpha (lane 3). Lane 4 shows DNA fragment molecular weight standard. (DOCX) [file pone.0182369.s010.docx]

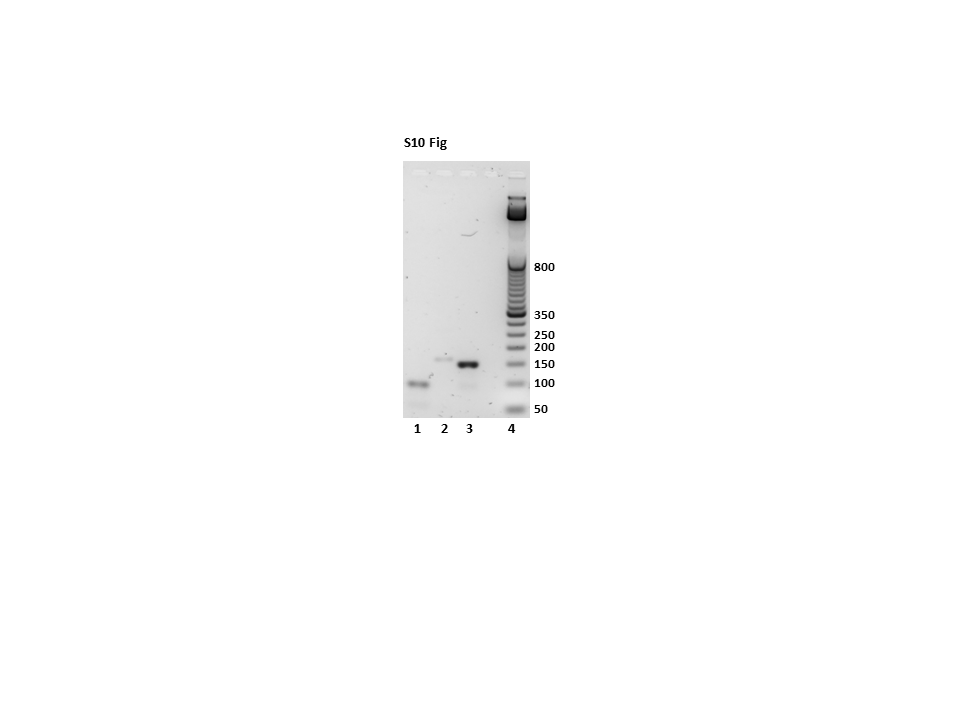


**S10 Fig - Expression of alveolar type (AT) I markers in L2 alveolar epithelial cells.**

RNA was isolated, reverse transcribed and the PCR products of the cDNA were separated by gel electrophoresis. DNA fragments were synthesized by PCR with primers for the AT I specific genes caveolin-1 (*lane 1*), receptor for advanced glycation end products (*lane 2*) and T1-alpha (*lane 3*). *Lane 4* shows DNA fragment molecular weight standard.
