# Supplementary material for: Variable stretch reduces the pro-inflammatory response of alveolar epithelial cells
Source: PLoS One. 2017 Aug 15;12(8):e0182369. doi: 10.1371/journal.pone.0182369 (PMC5557541; doi:10.1371/journal.pone.0182369)
Supplement: S11 Fig — RNA was isolated, reverse transcribed and the PCR products of the cDNA were separated by gel electrophoresis. DNA fragments were synthesized by PCR with primers for the AT II specific genes surfactant protein-A (lane 1), surfactant protein-B (lane 2), surfactant protein-C (lane 3) and surfactant protein-D (lane 4). Lane 5 shows DNA fragment molecular weight standard. As positive control served cDNA of rat lung tissue. (DOCX) [file pone.0182369.s011.docx]

**
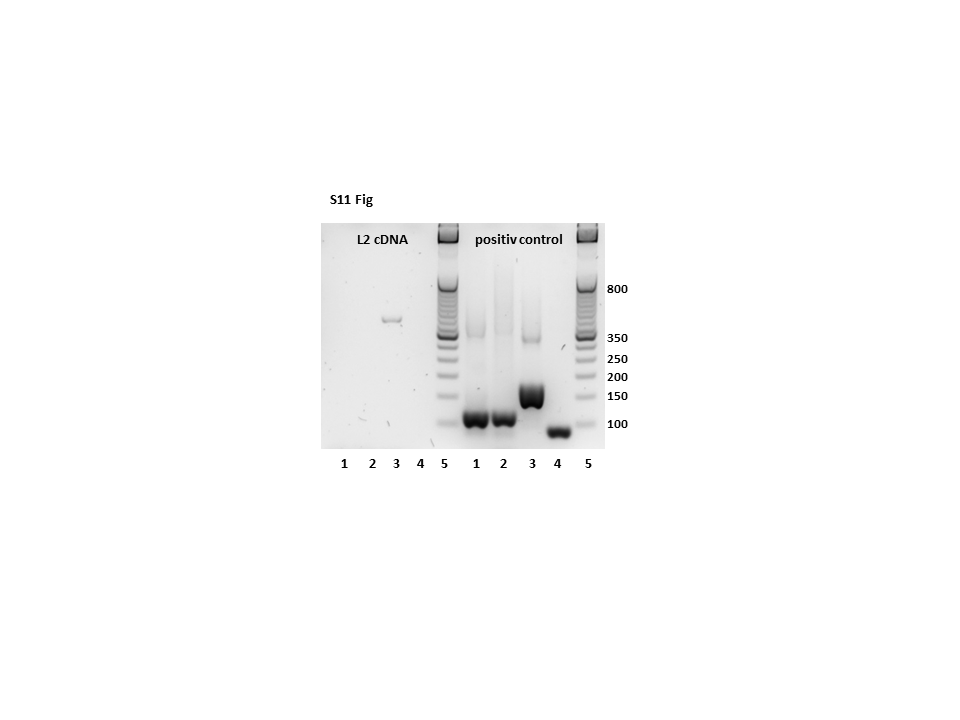
**

**S11 Fig - Expression of alveolar type (AT) II markers in L2 alveolar epithelial cells.**

RNA was isolated, reverse transcribed and the PCR products of the cDNA were separated by gel electrophoresis. DNA fragments were synthesized by PCR with primers for the AT II specific genes surfactant protein-A (*lane 1*), surfactant protein-B (*lane 2*), surfactant protein-C (*lane 3*) and surfactant protein-D (*lane 4*). *Lane 5* shows DNA fragment molecular weight standard. As positive control served cDNA of rat lung tissue.
